# P-693. The Influence of Covid-19 Pandemic on Respiratory Syncytial Virus Immunization in Alahsa Region: Cross-Sectional Study

**DOI:** 10.1093/ofid/ofaf695.906

**Published:** 2026-01-11

**Authors:** Mansoor Mishal Alnaim

**Affiliations:** National Guard Hospital, Hofuf, Ash Sharqiyah, Saudi Arabia

## Abstract

**Background:**

Respiratory Syncytial Virus vaccination is the primary control measure for severe complications caused by Respiratory Syncytial Virus infection. Moreover, Saudi Arabia recommends vaccinating people at risk against Respiratory Syncytial Virus to minimise co-infection risk with SARS-CoV2. Therefore, this study aims to assess the Saudi population's knowledge, attitude, and practice toward Respiratory Syncytial Virus vaccination during the COVID-19 pandemic. Moreover, we evaluate the impact of the COVID-19 pandemic on Respiratory Syncytial Virus vaccination.

**Methods:**

This cross-sectional study was conducted using an online survey in AlAhsa region between September 2024 to February 2025. Participants were invited to complete the questionnaire through a survey link sent to social media platforms.

**Results:**

A total of 1276 participants were included in this study. Our data demonstrate a lack of practice, attitude, and knowledge, especially on the Respiratory Syncytial virus's symptoms, viral transmission, and vaccine efficacy. Moreover, this study showed that the COVID-19 pandemic significantly impacted Respiratory Syncytial Virus vaccination in the AlAhsa region population by 1.2-times compared to the previous years.

**Conclusion:**

COVID-19 pandemic has increased the hesitancy of participants in Respiratory Syncytial Virus vaccination due to the lack of knowledge. As the pandemic of COVID 19 is fading, awareness campaigns are needed to encourage the public about the importance of receiving the Respiratory Syncytial Virus vaccination vaccine, especially for those at high risk for infection.

**Disclosures:**

All Authors: No reported disclosures

